# Grade 2 Disability in Leprosy in the Post-Elimination Era: A Case Report Highlighting Stigma and Programmatic Blind Spots

**DOI:** 10.7759/cureus.104011

**Published:** 2026-02-21

**Authors:** Ezhilarasan Selvaraju

**Affiliations:** 1 Community Medicine, Bhaarath Medical College and Hospital, BIHER (Bharath Institute of Higher Education and Research) University, Chennai, IND

**Keywords:** disability evaluation, disease eradication, elimination, leprosy, public health surveillance, social stigma

## Abstract

Leprosy has achieved elimination targets in many countries; however, patients continue to present with established disability. The presence of grade 2 disability (G2D) at diagnosis reflects delayed detection and persistent gaps in surveillance. I report a family cluster of three leprosy cases identified during routine program monitoring in Telangana, India, indicating a localized transmission pocket despite elimination status. The index case, a 35-year-old male with multibacillary leprosy, presented with advanced disability, including clawing of the right hand, plantar ulceration, and bilateral lagophthalmos. A structured G2D investigation revealed an overall diagnostic delay of approximately four years, including patient delay, provider delay associated with informal care, and missed detection during three consecutive rounds of leprosy case detection campaigns. Stigma further limited acceptance of home-based follow-up. Screening of four household contacts identified two children with paucibacillary leprosy without disability, suggesting earlier detection. The patient was managed according to national strategic plan guidelines, enrolled under the disability prevention and medical rehabilitation (DPMR) program, and provided multidisciplinary and community-based rehabilitation support. This case underscores the ongoing risk of preventable disability in the post-elimination era and highlights the need for structured G2D case audits, quality assurance of active case detection campaigns, strengthened surveillance, and stigma-sensitive community engagement to prevent delayed diagnosis in endemic pockets.

## Introduction

Leprosy continues to be reported from more than 120 countries, with approximately 200,000 new cases detected each year globally [1]. Although elimination as a public health problem, defined as a prevalence of fewer than one case per 10,000 population, was achieved worldwide in 2000 and in India in 2005, the disease persists and continues to cause clinical and social consequences [2].

At the national level, India reported a prevalence rate of 0.57 per 10,000 population, a grade 2 disability (G2D) rate of 1.31 per million population, and an annual new case detection rate (ANCDR) of 7.00 per 100,000 in 2024-25, underscoring ongoing transmission and delayed detection despite achieving elimination targets [2]. The continued occurrence of G2D among newly detected cases remains a sentinel indicator of diagnostic delay and program performance.

District-level heterogeneity further illustrates these challenges. District NLEP surveillance data for 2020-2021 indicate that Nirmal district reported a prevalence rate of 0.68 per 10,000 population, higher than several other districts in Telangana during the same period, while documenting zero grade 2 disability cases (District NLEP Office, Telangana, unpublished program data, 2021). Such variations suggest that prevalence-based elimination indicators alone may obscure localized gaps in early detection and disability surveillance.

Prevalence-based elimination targets may mask persistent deficiencies in surveillance intensity, particularly where programmatic momentum declines after elimination is achieved. Social stigma, limited community awareness, and reduced clinical suspicion among frontline healthcare providers further contribute to delayed presentation and progression to disability. Recognizing these challenges, India’s National Strategic Plan for Leprosy (2023-2027) aims to achieve zero transmission, zero disability, and zero discrimination [3]. However, the identification of new cases with established disability indicates the need for sustained surveillance and focused district-level attention.

This report describes a family cluster of leprosy detected during routine monitoring in Telangana, India, highlighting delayed diagnosis, stigma-related barriers, and the importance of structured G2D case investigation and quality assurance of leprosy case detection campaigns (LCDC).

## Case presentation

During routine district-level program monitoring in the Primary Health Centre area of Nirmal district, Telangana, India, a family cluster of leprosy was identified. The index case was a 35-year-old male diagnosed with multibacillary leprosy and initiated on multidrug therapy in 2022.

**Figure 1:**
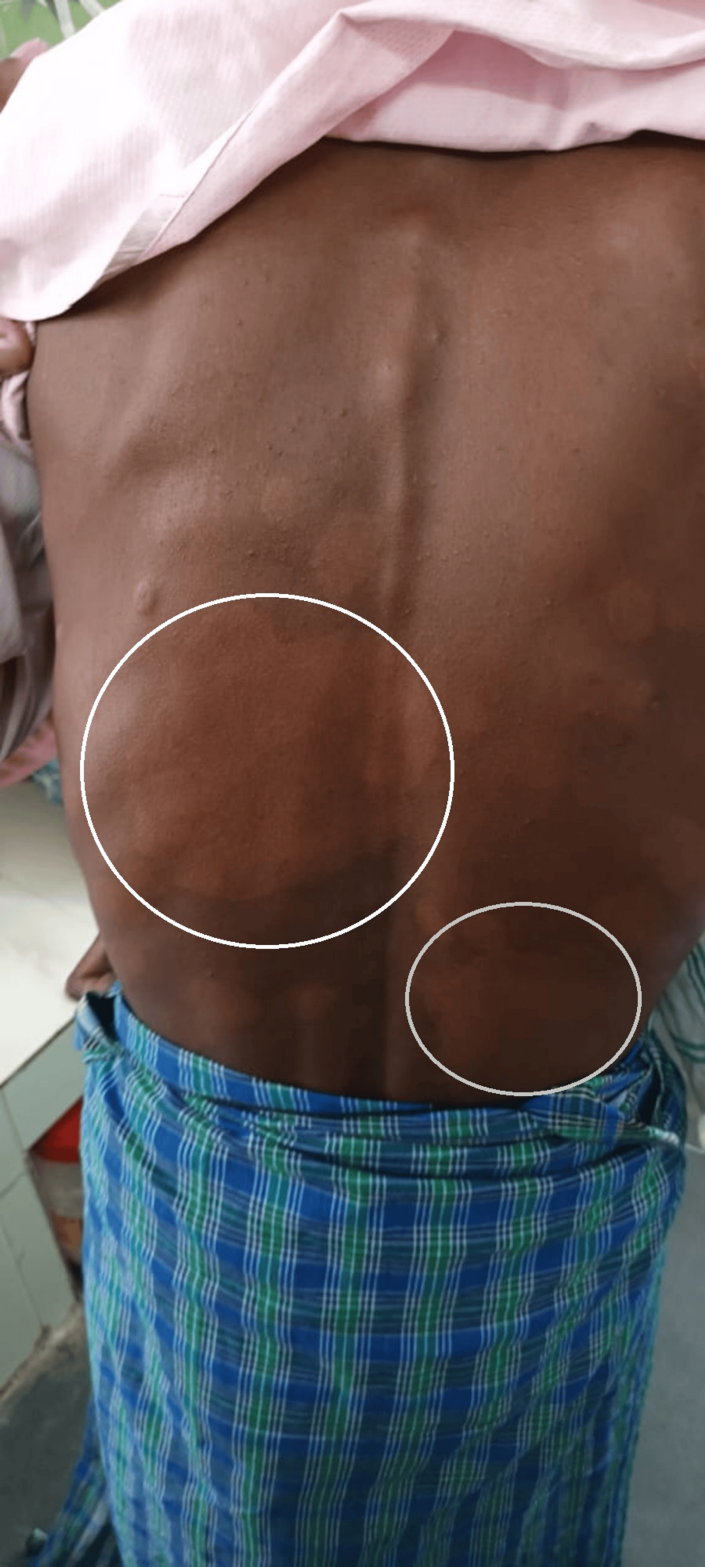
Well-defined hypopigmented anesthetic patch over the back consistent with leprosy.

On detailed examination, a well-defined hypopigmented anesthetic patch was noted over the back (Figure 1). 

**Figure 2:**
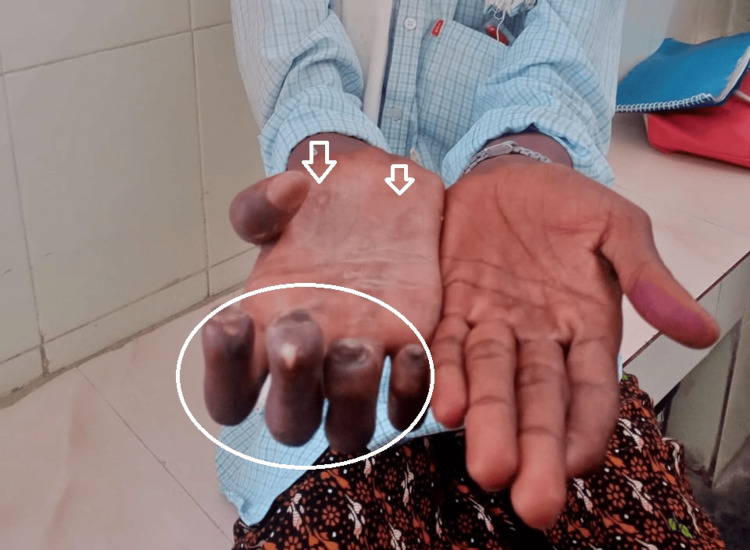
Grade 2 disability of the right hand demonstrating clawing of the fingers (circled) and wasting of the thenar and hypothenar eminences (arrow marked).

The right ulnar nerve was thickened, with sensory loss in the ulnar distribution of the right hand. Motor examination revealed clawing of the fingers with intrinsic muscle wasting of the right hand (Figure 2).

**Figure 3:**
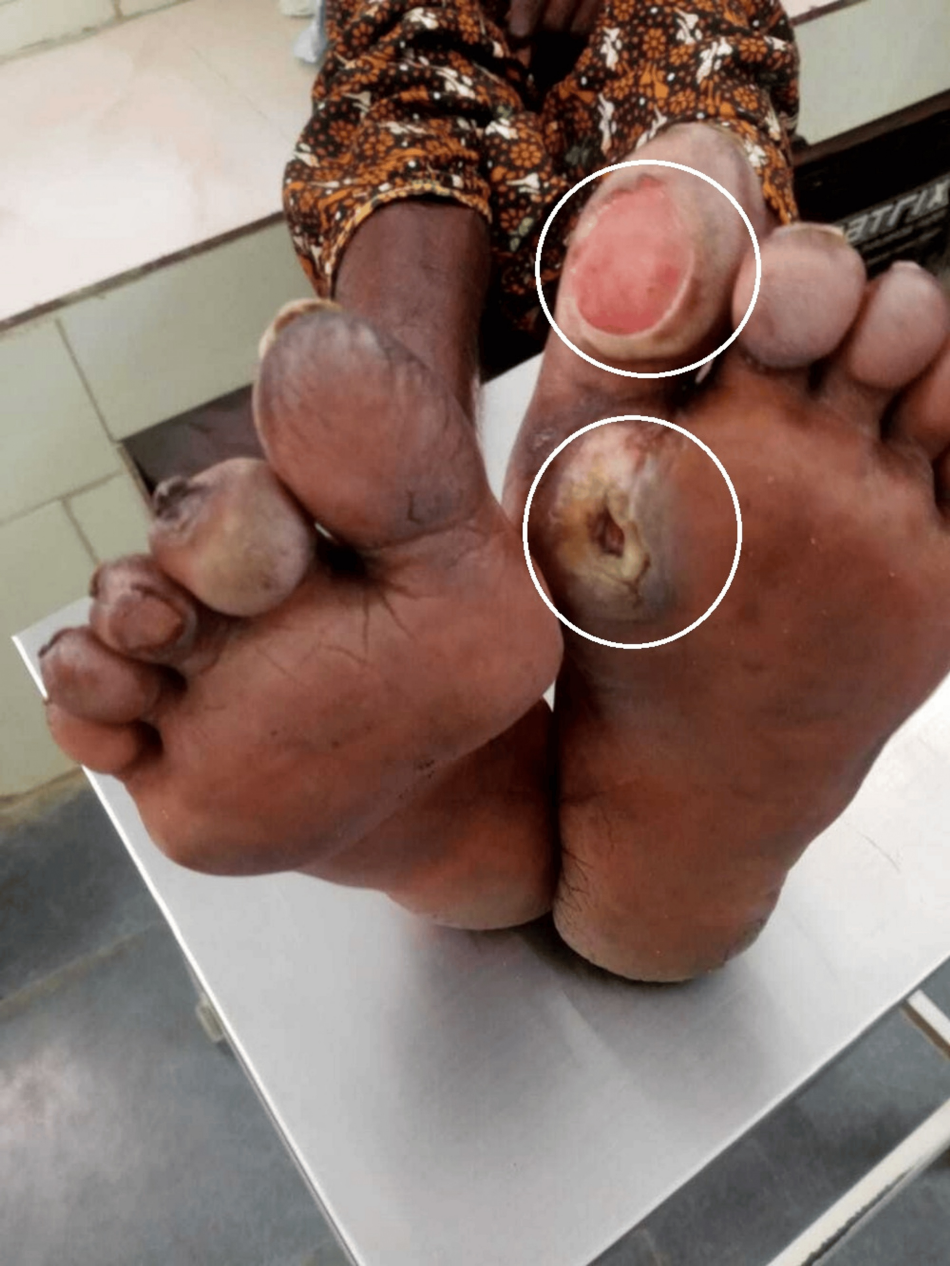
Plantar ulcer involving the left great toe associated with leprosy-related neuropathy.

A plantar ulcer involving the left great toe, consistent with neuropathic ulceration, was observed (Figure 3). 

**Figure 4:**
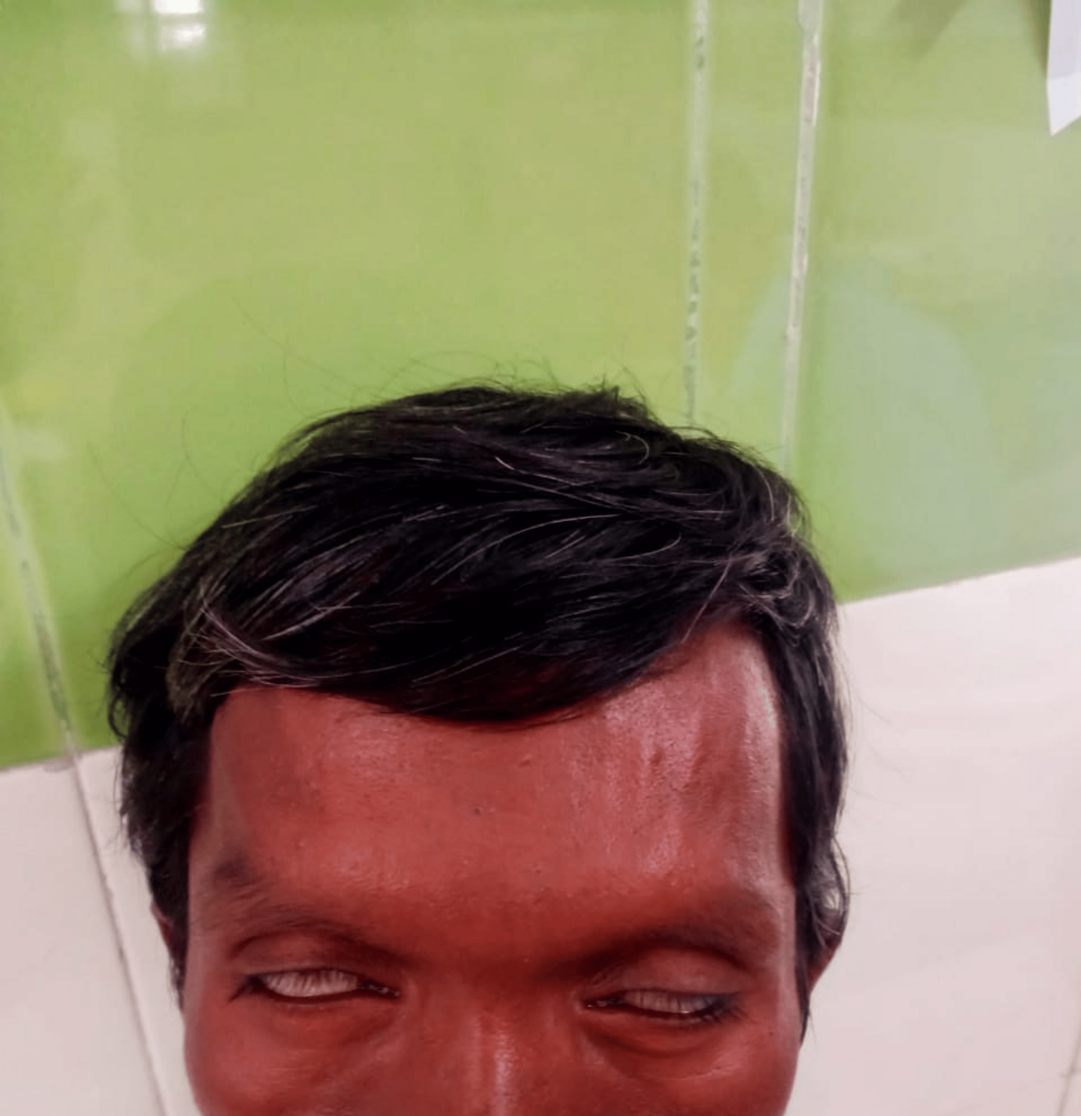
Bilateral lagophthalmos showing incomplete eyelid closure on attempted eye closure, consistent with facial nerve involvement in leprosy.

Ocular examination demonstrated bilateral lagophthalmos with reduced visual acuity (Figure 4).

Screening of four household contacts was conducted, among whom two children were diagnosed with paucibacillary leprosy later the same year. Neither child had evidence of disability at diagnosis, suggesting earlier detection compared to the index case.

A structured grade 2 disability (G2D) investigation revealed significant delays across the care pathway. The patient first noticed hypopigmented skin lesions approximately four years prior to diagnosis. There was an initial patient delay of nearly one year before he sought care from a local informal practitioner, where he received only symptomatic treatment without referral. This was followed by an additional delay of approximately two years at the provider and system levels before he accessed a government health facility for appropriate evaluation. During this period, he was not identified in three consecutive rounds of leprosy case detection campaigns (LCDC). The overall duration from symptom onset to initiation of multidrug therapy was approximately four years, reflecting substantial missed opportunities for early detection and prevention of disability.

Despite repeated counseling, the patient declined home visits by health workers due to fear of stigma and potential disclosure within the community. He preferred monthly facility-based visits for supervised multidrug therapy.

He was evaluated for corticosteroid therapy and managed for neuritis according to the National Strategic Plan guidelines [3], with supervised tapering. He was referred to the Ophthalmology Department at Government Medical College, Nirmal, and to the District Prevention of Disability and Medical Rehabilitation (DPMR) clinic, Nirmal. Monthly follow-up visits at the DPMR clinic focused on ulcer care, disability management, and eye protection. Accredited Social Health Activists (ASHAs) were sensitized to conduct weekly visits to reinforce adherence to self-care practices.

As part of comprehensive disability prevention, regular nerve function assessments (NFA) were initiated and monitored using eye-hand-foot (EHF) scoring. Given the presence of neuritis and lagophthalmos, the patient was kept under close surveillance with periodic reassessment. Counseling was provided to both the patient and his family regarding the cause of disability and protective measures for the hands, feet, and eyes. Self-care techniques, including ulcer management with emphasis on wound hygiene, protection, and monitoring of healing, were demonstrated and reinforced during follow-up visits. He was supplied with dressing materials and advised on the use of customized microcellular rubber (MCR) footwear to prevent further injury to anesthetic feet. Referral pathways for reconstructive services were also discussed as part of long-term rehabilitation planning. Psychosocial support and livelihood rehabilitation were facilitated with assistance from The Lepra Society and local non-governmental organization (NGO) partners.

Following identification of the family cluster, a focused leprosy campaign was conducted in the village with the participation of medical officers, auxiliary nurse midwives, and ASHA workers. Although only two suspects were identified and subsequently ruled out, the campaign highlighted gaps in community awareness and health-seeking behavior.

## Discussion

This case illustrates how leprosy-related stigma and diminished programmatic intensity in the post-elimination era can contribute to delayed diagnosis and preventable disability. The presence of grade 2 disability (G2D) at the time of diagnosis suggests that the disease had progressed for several years before appropriate treatment was initiated. Analysis of the care pathway revealed three interconnected components of delay: patient delay of approximately one year before seeking formal care, provider delay due to reliance on informal treatment without referral, and system-level gaps reflected in missed detection during earlier rounds of leprosy case detection campaigns (LCDC). Together, these factors highlight missed opportunities for timely identification and intervention at multiple levels of the health system.

The patient’s dependence on informal providers, non-detection during active surveillance activities, and reluctance to accept home-based follow-up underscore the influence of social stigma and structural barriers rather than individual negligence. G2D among newly detected cases is widely recognized as a sentinel indicator of delayed case detection and program performance. Similar patterns have been reported from Visakhapatnam district, India, where higher-than-national-average G2D prevalence persisted despite implementation of case detection campaigns [4].

Qualitative evidence from Telangana further highlights the influence of stigma, gender norms, family dynamics, and communication gaps in delaying diagnosis and contributing to disability [5]. Broader discussions on leprosy elimination have raised concerns that early achievement of prevalence targets may reduce political and programmatic attention, allowing G2D to persist as a marker of delayed detection [6].

In addition to clinical management, long-term rehabilitation and social reintegration remain essential components of care. The patient was encouraged to participate in community-based self-help groups for persons affected by leprosy and disability. Evidence from rural India suggests that such groups can remain functional even after withdrawal of donor support and may contribute to economic stability, social inclusion, and reduction of stigma [7]. These community-based mechanisms may help mitigate the psychosocial and economic consequences of disability, particularly in settings where stigma limits participation in routine community life.

The identification of three cases within a single household also suggests a localized pocket of transmission. Such clusters emphasize the need for systematic contact screening, high-quality LCDC implementation, and ongoing surveillance even in areas that have achieved elimination thresholds. Monitoring G2D trends, strengthening frontline clinical training, engaging informal care providers, and addressing stigma at the community level remain critical to preventing avoidable disability.

## Conclusions

This case underscores that achieving elimination targets does not necessarily translate into timely diagnosis or prevention of disability. The presence of grade 2 disability (G2D) at diagnosis reflects prolonged delays influenced by stigma, reliance on informal care, and gaps in active case detection efforts. The identification of a family cluster further indicates the possibility of ongoing localized transmission despite elimination status.

To address these gaps, two immediate programmatic actions are warranted. First, institutionalization of structured G2D case audits at the district level can enable systematic analysis of diagnostic delays and identification of missed opportunities within the care pathway. Second, periodic independent quality assurance reviews of leprosy case detection campaigns (LCDC) are essential to ensure effective coverage and early detection. In addition, engagement of informal healthcare providers through targeted sensitization programs and integration of stigma-reduction messaging into village health platforms may help reduce patient and provider delays. Strengthening surveillance systems, reinforcing systematic G2D investigations, and promoting stigma-sensitive community engagement remain critical to preventing avoidable disability and sustaining progress toward zero transmission, zero disability, and zero discrimination.
